# Effect of Steam Blanching and Drying on Phenolic Compounds of Litchi Pericarp

**DOI:** 10.3390/molecules21060729

**Published:** 2016-06-03

**Authors:** Honest N. E. Kessy, Zhuoyan Hu, Lei Zhao, Molin Zhou

**Affiliations:** Department of Food Engineering, College of Food Science, South China Agricultural University, 483, Wushan Road, Guangzhou 510642, China; signage2000@ymail.com (H.N.E.K.); scauzl@scau.edu.cn (L.Z.); tianruojinxi@163.com (M.Z.)

**Keywords:** litchi pericarp, blanching, drying, phenolic compounds, antioxidant activity

## Abstract

The effects of different treatment methods on the stability and antioxidant capacity of the bioactive phenolic compounds of litchi pericarps were investigated. Fresh litchi pericarps were open air–dried, steam-blanched for 3 min in combination with hot air oven drying at 60 and 80 °C, and unblanched pericarps were dried in a hot air oven at 40, 60, 70 and 80 °C until equilibrium weight was reached. The total phenolic compounds, flavonoids, anthocyanins, proanthocyanidins and individual procyanidins, and antioxidant activity were analyzed. The combination of blanching and drying at 60 °C significantly (*p* < 0.05) improved the release of phenolic compounds, individual procyanidins, and the extracts′ antioxidant capacity compared with the unblanched hot air oven-dried and open air–dried pericarps. Drying of fresh unblanched litchi pericarps in either open air or a hot air oven caused significant losses (*p* < 0.05) in phenolic compounds and individual procyanidins, leading to a reduction in the antioxidant activity. A similar increase, retention or reduction was reflected in flavonoids, proanthocyanidins and anthocyanins because they are sub-groups of phenolic compounds. Ferric reducing antioxidant power (FRAP) and 1,1-diphenyl-2-picryldydrazyl (DPPH) radical-scavenging capacity of the treated pericarps were significantly correlated (*r* ≥ 0.927, *p* < 0.01) with the total phenolic compounds. Thus, the combination of steam blanching and drying treatments of fresh litchi pericarps could produce a stable and dry litchi pericarp that maintains phenolic compounds and antioxidant capacity as a raw material for further recovery of the phytochemicals.

## 1. Introduction

Litchi processing such as wine and juice production generates large amounts of peels/pericarps, seeds and pulp as by-products, where the bioactive phenolic compounds are mostly concentrated [1,2,3]. The litchi pericarp is credited as a potential source of health-promoting bioactive phenolic compounds and as an ingredient in the formulation of oligonol, a low molecular weight proanthocyanidin supplement [3]. However, despite litchi pericarps being composed of a high content of bioactive phenolic compounds, they are highly perishable due to the high activity of endogenous enzymes such as polyphenol oxidases (PPO) and peroxidase (POD) which catalyze the oxidation of the phenolic compounds [4,5]. This factor can hinder utilization of litchi pericarps in the recovery of phenolic compounds, especially during peak harvest and processing season if no initiatives are taken for the treatment of the pericarps for further utilization.

During juice and wine processing of litchi, the by-products are piled up, mixed, and they generate heat as a result of biochemical processes, elevating the degradation of the bioactive compounds. Therefore, after separation of the pericarps from the aril, the pericarps should be treated within the shortest time possible to preserve the bioactive phenolic compounds. The storage of fresh raw materials is a challenge in agro-processing industries. Blanching (such as steam blanching) and drying (such as open air, hot air oven, and freeze drying) treatments are common post-harvest methods employed to extend the shelf life of raw materials or final products. Studies on grape pomace [6], herbs [7,8] and apple pomace [9] show that various treatment methods and conditions have different influences on the phytochemicals in various materials. Hence, understanding the roles of blanching and drying in impacting the bioactive phenolic compounds and selecting appropriate treatment methods and conditions is of significant importance in the utilization of litchi pericarps.

This study evaluated the effects of steam blanching and oven drying under different conditions on the phenols, flavonoids, proanthocyanins, anthocyanins and procyanidin monomers in litchi pericarps. Various combinations of branching and air drying were applied, including combined steam blanching and hot air oven drying (at 60 or 80 °C) as well as open air and hot air oven drying of unblanched litchi pericarps at different temperatures (40, 60, 70 and 80 °C). An appropriate pre-treatment method was then suggested for enhancing the release and preservation of phenolic compounds. The results of this study will provide guidelines for the selection of pretreatment methods for producing litchi pericarps with maximum phenolic compounds and antioxidants.

## 2. Results and Discussion

A fresh litchi pericarp contains 59.57% ± 0.59% of moisture and 40.43% ± 0.61% of dry matter which coincided with results previously reported by Li *et al.* [10]. Combined steam blanching and hot air oven drying as well as open air drying reduced the moisture contents to 10.81% ± 3.13% and 15.32% ± 4.42%, respectively, while after hot air oven drying the moisture content ranged between 10.91% ± 2.61% to 12.86% ± 3.54%. The water content of the litchi pericarp was reduced below the level which is critical for post-harvest storage of plant materials. Open air drying requires several days depending on the weather, while hot air oven drying requires only a few hours (high or low temperatures) to attain the same water content during the drying process.

### 2.1. Total Polyphenols, Flavonoids, Proanthocyanidins, and Anthocyanins Contents

The effect of various treatment methods on the phenolic compounds of litchi pericarp is illustrated in Table 1. It was observed that both steam blanching and drying processes significantly (*p* < 0.05) influenced the stability of total phenolic compounds, flavonoids, proanthocyanidins and anthocyanins. The total phenolic compounds, flavonoids, proanthocyanidins and anthocyanins were reduced by 26.44%, 15.11%, 14.12% and 78.61% after open air drying for seven days. Hot air oven drying at 40 °C decreased the total phenolic, flavonoids and anthocyanidins contents by 12.03%, 18.38% and 60.01%, respectively, whereas drying above a temperature of 60 °C leads to a reduction of the phenolic groups by over 40%.

The combination of steam blanching and hot air oven drying (60 °C) retained and significantly improved the release of the phenolic compounds. The release of flavonoids was enhanced by 14.35% and proanthocyanidins were increased by 27%. However, hot air oven drying (at 80 °C) of the blanched pericarps caused thermal degradation of phenolic compounds, flavonoids, proanthocyanidins and anthocyanidins, with the amounts reduced by 49%, 34.23%, 41.15% and 72.77%, respectively. Open air drying, hot air oven drying at 40 °C and hot air oven drying at 60 °C caused significant degradation of the phenolic compounds at lower rates compared with the hot air oven drying at above 70 °C. However, open air–dried and hot air oven-dried (at 70 °C) samples of pericarps had similar loss rates of anthocyanins (>50%), which indicated anthocyanins of litchi pericarps are highly susceptible to enzymes catalyzed during oxidation and high temperature treatments.

The improved release of phenolic compounds observed in combination steam-blanched and hot air oven-dried pericarps might be attributed to the inactivation of oxidative enzymes and the induced structural changes in the pericarps’ cell matrices, leading to improved release of extractable and non-extractable phenolic compounds [11]. Degradation of phenolic compounds of litchi pericarps has been associated with activities of endogenous enzymes such as PPO, POD, and anthocyanase which catalyze the oxidation of the phenolic compounds [12]. Ambient temperatures could favor high activities of these enzymes while high temperatures could cause loss of phenolic compounds through both thermal degradation and enzymatic oxidation.

Similar increase or retention was reflected in flavonoids, proanthocyanidins, anthocyanins and individual procyanidin monomers because they are sub-groups of phenolic compounds. A previous study by Heras-Ramirez *et al.* [9] reported that blanching enhanced the release of phenolic compounds and the antioxidant activity of apple pomace polyphenols, while hot air drying treatments caused significant losses in total phenolic compounds, flavonoids, proanthocyanidins, and anthocyanins.

Hot air oven drying at temperatures exceeding 60 °C showed a drastic loss of total phenolic compounds with a similar trend observed in anthocyanins, proanthocyanidins, and flavonoids. The observed loss may be associated with the effect of high temperatures, which accelerate the degradation of the phenolic compounds. Previous literature also reported significant losses in total phenolic compounds, proanthocyanidins, and anthocyanidins in grape pomace, mulberry leaves, and carrots, caused by thermal degradation at high hot air oven-drying temperatures [6,9,13,14].

### 2.2. Identification of Litchi Procyanidins

**Figure 1:**
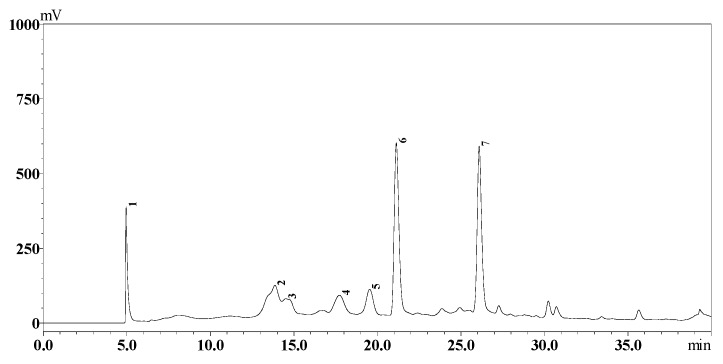
HPLC chromatogram pofiles of major procyanidins of litchi pericarp.

HPLC fingerprints showed the presence of procyanidins in the extract (Figure 1) which were tentatively identified as procyanidin B2 (peak 4), epicatechin (peak 6), procyanidin A2 (peak 7) and catechin (peak 5) by relating the retention time to the standard compounds. In the MS spectrum, compound peak (4) produced molecular ion [M − H]**^−^** at *m*/*z* 577.2 and two fragment ions at *m*/*z* 289.1 and 407. According to external standards, these compounds were confirmed as procyanidins B-type (B1 or B2 dimmers). The compound peaks 5 and 6 exhibited a signal at [M − H]^−^ at *m*/*z* 289.1 and a fragment ion at 245.1, and they were identified as epicatechin or catechin according to the external standard. Compound peak 7 produced a molecular ion ([M − H]^−^) at *m*/*z* 575.2 and the fragment ion at 285.1 was confirmed as procyanidin A2 using external standard and based on a previous reference [15].

### 2.3. Effect of Treatment on Procyanidins

Litchi (*Litchi chinensis* Sonn. cv Huaizhi) is usually utilized for juicing and wine-making. Its pericarps constitute significant amounts of phenolic compounds, flavonoid, proanthocyanidin and anthocyanin contents, thus potential raw materials for recovery phenolic compounds. Therefore, it was selected for evaluation of the effect of the combination of steam blanching and drying methods on the individual procyanidin profiles as shown in Table 2. The procyanidin A2 and epicatechin were the major procyanidins, which was in agreement with previous literature [16,17].

The individual procyanidins were reduced significantly (*p* < 0.05) by drying conditions. Open air drying reduced the content of procyanidins by 30% to 57%. Epicatechin and catechin showed the highest degradation levels of 41% and 57%. Hot air oven drying above 40 °C caused degradation of procyanidin A2 (51%), procyanidin B2 (56%), epicatechin (47%) and catechin (51%). The combination of steam blanching and hot air oven drying at 60 °C significantly increased the concentration of individual procyanidins (*p* < 0.05), probably caused by the inactivation of enzymes such as PPO and POD and the enhanced release of bound procyanidins from pericarp matrices [18]. The improved recovery of epicatechin, procyanidin A2, and procyanidin B2 contents were 18.55%, 14.26%, and 12.42%, respectively. Degradation of epicatechin, procyanidin A2 and procyanidin B2 contents by 50%, 42%, and 57% was observed in blanched and hot air oven-dried pericarps at 80 °C, respectively.

The combination of temperature, the long drying time, and PPO and POD activity may have been the main factors causing the degradation of the individual procyanidins. Open air drying is a lengthy process at an ambient temperature which favors the enzyme catalyzing the oxidation of procyanidin monomers and anthocyanins, leading to the formation of brown polymeric pigments [19]. Sun *et al*. [20] reported procyanidin A2 and (−)-epicatechin as the substrate to PPO enzyme-catalyzed oxidation, leading to their degradation during dehydration. Khanal *et al.* [21] investigated effects of heating on procyanidin and anthocyanin stability in grape and blueberry pomace. Their findings showed that procyanidin and anthocyanin contents were significantly degraded at high heating temperatures, which seems to be the same trend in our findings. The capacity of procyanidins to depolymerize or bind to plant cell wall polysaccharides during the drying process may also reduce the content of the extractable procyanidins [22]. Comparing the results observed of open air and hot air oven drying at 40 °C, procyanidin monomers were susceptible to high thermal degradation or combination of both thermal and enzymatic oxidation. Generally, drying processes could induce undesirable effects on the constituent profiles of plant phytochemicals. Therefore, caution should be taken when employing drying methods and conditions in pre-treatment of plant materials destined for the recovery of bioactive compounds [23]. Thus, the combination of steam blanching and hot air oven drying at 60 °C could preserve litchi pericarps as raw materials for further recovery of the bioactive phenolic compounds.

### 2.4. Effect of Treatment on Total Antioxidant Activity

**Figure 2:**
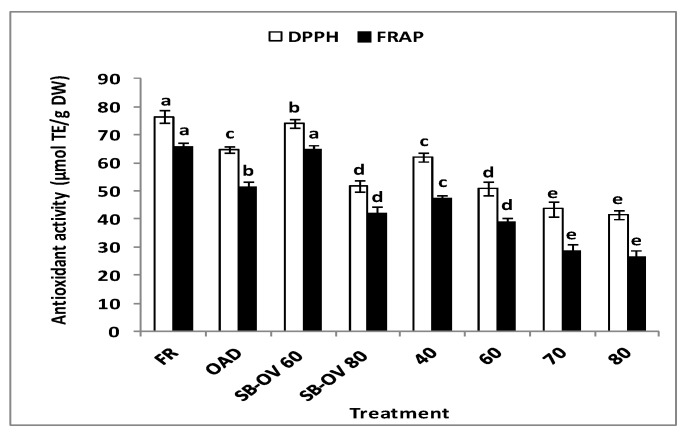
Effect of treatment condition on the antioxidant capacity of litchi pericarps evaluated by DPPH and FRAP assays. (FR: Fresh pericarp, OAD: Open air drying, SB-OV: Combined steam blanching and hot air oven drying. Means within a series between the bars with similar letters were not significantly different (*p* > 0.05). Data were mean ± SD, *n* = 3).

1,1-diphenyl-2-picryldydrazyl (DPPH) and ferric reducing antioxidant power (FRAP) assays were employed to measure the antioxidant capacity of extracts. The latter is based on the ability of antioxidants to behave as radical scavengers while the former determines the ability of antioxidants to act as reducing agents [24]. Total antioxidant capacity was significantly associated with blanching and drying temperatures (Figure 2). FRAP and DPPH radical-scavenging capacity of the treated pericarps were significantly correlated (*r* > 0.927, *p* < 0.01) with the total phenolic compounds. Strong correlation of phenolic compounds with antioxidant activity was previously reported in litchi pericarps [10,25] and in berries [26]. In general, open air and hot air oven drying treatments reduced the antioxidant capacity of the pericarps, which corresponded to the high degradation of the bioactive phenolic compounds observed. FRAP and DPPH radical-scavenging capacity of the pericarps dried at temperatures above 60 °C decreased significantly at an average of 52.53% and 25.55%. Steam-blanched and hot air oven-dried (60 °C) pericarps exhibited improved FRAP and DPPH antioxidant activity by 13.08% and 9.84%, as was expected from the enhanced release of the phenolic compounds.

The treatment method showed a significant correlation with the phenolic compounds content (*r* = 0.697, *p* < 0.01), indicating that treatment methods and conditions influenced the stability of phenolic compounds, and thus the antioxidant activity. A similar trend in the reduction of antioxidant capacity and phenolic content was observed when different drying treatments were employed on apple pomace [9], grape pomace [6] and herbs [24].

## 3. Materials and Methods

### 3.1. Sample Preparation and Treatment

Litchi fruits (*Litchi chinensis* Sonn. cv Huaizhi) at commercial mature stage were from orchards in Guangdong Province. The fruits were selected based on the uniformity of size and color, and were then washed with chilled water, peeled and the pericarps were immediately divided into batches of 2 kg each in three sets. Batches were assigned treatments as the combination of steam blanching at for 3 min and hot air oven drying at 60 and 80 °C, open air drying, hot air oven drying (40, 60, 70 and 80 °C). Samples were dried until equilibrium weight was reached. The dried pericarps were ground to fine powder with a stainless-steel grinder (particle size 2 mm) and kept at room temperature until use. Fresh samples were immediately dried in liquid nitrogen; ground and extracted. Fresh samples were set as a control sample. The drying methods and steam blanching conditions employed are shown in Table 3. The moisture (%) of treated litchi pericarp was calculated according to Katsube *et al*. [13]. In brief, treated litchi pericarps moisture content (%) was determined by measuring change in weight before and after drying in the convection oven at 105 °C for 5 h. The dry matter in the fresh litchi pericarps was determined by calculating percentage of the weight remaining after drying fresh litchi pericarps at 105 °C for 5 h.

### 3.2. Chemicals

Methanol, acetonitrile, and acetic acid were from Merck, Germany, with 98% purity, HPLC grade. Gallic acid, procyanidin A2, proanthocyanidins, vanillin, (+)-catechin (C), cyanidin-3-glucoside, procyanidin B2, (−)-epicatechin (EC), 6-hydroxy-2,5,7,8-tetramethylchroman-2-carboxylic acid (Trolox), Folin-Ciocalteu reagent, 1,1-diphenyl-2-picryldydrazyl (DPPH), tripyridyl-triazine (TPTZ) were supplied from Sigma-Aldrich (St. Louis, MO, USA). Other chemicals and solvents used were analytical grade from local suppliers (Sinopharm, Guangzhou, China).

### 3.3. Extraction of Phenolic Compounds

Litchi pericarps powder (1:3, *w*/*v*) were extracted in ethanol (60%) at 40 °C in a thermostatically temperature controlled orbital shaker water bath agitating (180 r/min) for 2 h. The residues were re-extracted twice; the supernatant was pooled and centrifuged at 2500 r/min for 20 min. The extract solution was dried in a rotary vacuum evaporator at 45 °C and reduced pressure to dryness. The extract was stored in a dark bottle at −18 °C till analysis.

### 3.4. Total Polyphenols and Flavonoids

Estimation of total phenolic compounds was determined by Folin-Ciocalteu reagent (FC, 1N, Sigma-Aldrich, St. Louis, MO, USA) according to the method of Dewanto *et al.* [27]. The total phenolic content was expressed as milligrams of gallic acid equivalent (GAE) on dry weight (DW) basis (mg GAE/g DW) from the calibration curve of the standard gallic acid. Total flavonoids were estimated by a colorimetric protocol using rutin as standard and expressed as mg RE/g DW [28].

### 3.5. Determination of Proanthocyanidins

Litchi pericarps (1:3, *w*/*v*) were extracted overnight by 70% acetone solution. The mixture was centrifuged at 2500 r/min for 20 min. The extract solution was dried in a rotary vacuum evaporator at 45 °C to remove the organic solvent. Proanthocyanidins were estimated using vanillin reagent according to Wang *et al.* [29] with some modification. Briefly, 2 mL of aqueous sample was dried and reconstituted in 2 mL of absolute methanol; 0.5 mL of samples was reacted with 3.5 mL of vanillin solution (0.5%) in 8% HCl in absolute methanol. The sample mixture was incubated in water bath at 30 °C for 20 min and absorbance recorded at 500 nm. A standard curve was constructed using authentic proanthocyanidins in mg/g DW.

### 3.6. Extraction and Determination of Anthocyanins

Five grams of litchi pericarps was extracted twice with 30 mL of ethanol containing 0.1 M HCl in a water bath set at 40 °C for 3 h. The content of the total anthocyanins was measured by the pH differential method [30]. In brief, the absorbance values of litchi extract (1:5, *v*/*v*) in 0.1 M KCl-HCl buffer (pH 1.0) solution and 0.4 M sodium acetate buffer (pH 4.5) were scanned at 515 nm and 700 nm. The total anthocyanins concentration was calculated using cyanidin-3-glucoside as reference standard as follows:

Anthocyanins content (mg/100 g) = A × MW × 1000/(ε × C)(1)

where A is absorbance difference = (A_515_ − A_700_) pH 1.0 − (A_515_ − A_700_) pH 4.5, MW and ε is the cyanidin-3-glucoside molecular weight (449.2) and molar absorptive (26900) and C is the concentration of the buffer concentration (mg/mL).

### 3.7. Procyanidins Profile Analysis

The analysis was performed on Shimadzu LC-20 (Shimadzu Corporation, Kyoto, Japan), ZOBRAX Eclipse XDB-C18 column, particle size 250 mm × 4.6 mm, 5 µm (Agilent Technologies Inc. Santa Clara, CA, USA) and an SPD-10A UV-VIS detector. The mobile phase consisted of 1% *v*/*v* aqueous acetic acid (mobile phase A) and absolute methanol (mobile phase B). Elution was performed with 10%–60% B for 35 min, 100% B for 10 min, and 10% B for 5 min at 30 °C, a flow rate of 0.5 mL/min, and a sample volume of 10 µL. The column was equilibrated with 10% B for 5 min before next injection. Tentative identification of phenolic compounds in the samples, external standards of (+)-catechin(C), (−)-epicatechin (EC), procyanidin A2, procyanidin B2 were used. For molecular weight identification, an LC-MS system, equipped with an ESI interface (Agilent Technologies Co., Ltd., Santa Clara, CA, USA) was used. Similar elution conditions as described above were followed. The procyanidins were detected by ESI-MS set at a negative ion mode. The orifice voltage was −30 V and heat capillary temperature was 275 °C. The mass scale was defined from 100 to 1200 *m*/*z*.

### 3.8. Determination of FRAP and DPPH Radical-Scavenging Activity

DPPH radical-scavenging activity was carried out according to the method suggested by Duan *et al*. [31] with modifications. Briefly, aliquots of samples 60 µL were added to 3.94 mL of DPPH in methanol (0.1 mM/L). The solution was mixed on vortex and incubated at 30 °C for 30 min protected from light. The absorbance was measured at 517 nm. Low values of absorbance indicated a high free radical scavenging activity. FRAP was performed following a procedure of Benzie *et al*. [32] with modifications. In brief, preparation of FRAP reagent solution was carried out by mixing acetate buffer (pH 3.6), TPTZ solution (10 mM) in 40 mM HCl and 20 mM iron (III) chloride solution (10:1:1, *v*/*v*). The reagent was daily prepared and pre-incubated in a water bath at 37 °C prior to use. A 200 µL of sample were mixed with 1.8 mL of the FRAP reagent. The mixture was incubated at 37 °C in the water bath for 10 min before recording absorbance at 593 nm. Both FRAP and DPPH were presented as µmol Trolox equivalents (TE)/g of dry weight from standard Trolox curve.

### 3.9. Statistical Analysis

One-way analysis of variance (ANOVA) and Duncan test was conducted for the separation of means using the SPSS 17.0.1 statistical package for Windows (SPSS Inc., Chicago, IL, USA). Results were expressed as means ± standard deviations (SD).

## 4. Conclusions

The present study shows that phenolic compounds of litchi pericarps are susceptible to degradation at high drying temperatures (>60 °C) and drying at ambient temperatures for a long duration. Open air drying leads to long-lived preservation and is a low-cost natural method. However, the lengthy drying period at ambient temperatures favors activities of the endogenous enzymes, leading to the oxidation of phenolic compounds. Among the phenolic compounds sub-groups, anthocyanins and procyanidin monomers were notably degraded in open air–dried litchi pericarps. Thus, for litchi pericarps destined for the recovery of anthocyanidin pigments or procyanidin monomers, open air drying may not be a suitable pre-treatment method. The combination of steam blanching and hot air oven drying treatments led to a higher recovery of the phenolic compounds and enhanced the antioxidant capacity. For better recovery of bioactive phenolic compounds from litchi pericarps, the combination of steam blanching and hot air oven drying (60 °C) as a pre-treatment may be favorable.

## Figures and Tables

**Table 1:** Influence of treatment on total phenolic compounds, flavonoids, proanthocyanidins and anthocyanidins of litchi pericarps.

Treatment	Total Phenolics (mg GAE/g)	Total Flavonoids (mg RE/g)	Total Procyanidins (mg/g)	Total Anthocyanins (mg CE/100g)
Fresh pericarp	72.61 ± 1.32 ^b^	33.14 ± 0.32 ^a^	25.52 ± 1.04 ^b^	25.06 ± 0.13 ^a^
Open air drying	53.41 ± 1.08 ^e^	28.13 ± 1.66 ^b^	21.89 ± 1.76 ^b^	5.36 ± 0.26 ^d^
SB-OV 60 °C	82.26 ± 1.12 ^a^	37.06 ± 0.19 ^a^	32.72 ± 0.92 ^a^	20.32 ± 0.14 ^b^
SB-OV 80 °C	39.26 ± 1.32 ^f^	29.05 ± 0.24 ^b^	14.68 ± 0.42 ^c^	7.25 ± 0.15 ^c^
Hot air oven 40 °C	63.87 ± 0.35 ^c^	28.04 ± 0.12 ^b^	24.17 ± 0.12 ^b^	10.02 ± 0.32 ^c^
Hot air oven 60 °C	59.68 ± 1.31 ^d^	28.15 ± 2.42 ^b^	20.24 ± 0.14 ^b^	6.19 ± 0.67 ^d^
Hot air oven 70 °C	37.62 ± 0.42 ^f^	19.69 ± 1.62 ^c^	15.37 ± 0.26 ^c^	5.53 ± 0.79 ^d^
Hot air oven 80 °C	35.72 ± 0.24 ^f^	18.08 ± 1.59 ^c^	16.20 ± 0.35 ^c^	5.03 ± 0.89 ^d^

Different letters within a column indicate means are significantly different (*p* < 0.05). SB-OV: combined steam blanching and hot air oven drying; GAE: gallic acid equivalents; RE: rutin equivalents; CE: cyanidin-3-glucoside equivalents. Data are expressed in dry weight (DW) basis. Values are mean ± standard deviation (*n* = 3).

**Table 2:** Effect of treatments on major procyanidins of litchi pericarps.

Treatment	Procyanidin A2 (mg/g DW)	Procyanidin B2 (mg/g DW)	Epicatechin (mg/g DW)	Catechin (mg/g DW)
Fresh pericarps	7.22 ± 0.46 ^b^	4.83 ± 0.16 ^b^	19.13 ± 1.04 ^b^	1.51 ± 0.03 ^a^
Open air drying	5.02 ± 0.52 ^c^	3.27 ± 0.22 ^c^	11.17 ± 0.33 ^c,d^	0.64 ± 0.04 ^c^
SB-OV 60 °C	8.25 ± 0.36 ^a^	5.43 ± 0.23 ^a^	22.68 ± 0.61 ^a^	1.42 ± 0.09 ^a^
SB-OV 80 °C	4.14 ± 0.28 ^d^	2.04 ± 0.11 ^e^	9.45 ± 1.66 ^d^	0.81 ± 0.02 ^b^
Hot air oven 40 °C	4.43 ± 0.16 ^c^	2.68 ± 0.09 ^d^	12.92 ± 0.13 ^c^	0.86 ± 0.06 ^b^
Hot air oven 60 °C	3.51 ± 0.36 ^d^	2.08 ± 0.04 ^e^	10.07 ± 0.48 ^d^	0.73 ± 0.05 ^c^
Hot air oven 70 °C	3.06 ± 0.26 ^d^	1.46 ± 0.09 ^f^	8.36 ± 0.24 ^e,f^	0.46 ± 0.03 ^e^
Hot air oven 80 °C	3.93 ± 0.19 ^d^	1.99 ± 0.08 ^e^	6.99 ± 0.28 ^f^	0.56 ± 0.05 ^d^

Values are means ± standard deviation (*n* = 2). Different letters in the column indicated significant different (*p* < 0.05). SB-OV: combination of steam blanching and hot air drying.

**Table 3:** Temperature, time and final moisture content of dried litchi pericarps.

Method	Temperature (°C)	Time	Final Moisture (%)
Open air drying	28–32	5–7 days	15.32 ± 4.42
Hot air oven Drying	40	8.0 h	12.86 ± 3.54
	60	5.5 h	12.29 ± 2.40
	70	3.0 h	11.73 ± 1.97
	80	2.0 h	10.91 ± 2.61
SB-OV	60	5.0 h	11.02 ± 3.04
SB-OV	80	2.0 h	10.59 ± 3.22

Values were expressed as the mean ± standard deviation (*n* = 3). SB-OV: combined steam blanching and hot air oven drying.
